# Shared Environment and Genetics Shape the Gut Microbiome after Infant Adoption

**DOI:** 10.1128/mBio.00548-21

**Published:** 2021-03-30

**Authors:** Hannah F. Tavalire, Diana M. Christie, Leslie D. Leve, Nelson Ting, William A. Cresko, Brendan J. M. Bohannan

**Affiliations:** a Prevention Science Institute, University of Oregon, Eugene, Oregon, USA; b Institute of Ecology and Evolution, University of Oregon, Eugene, Oregon, USA; c Department of Anthropology, University of Oregon, Eugene, Oregon, USA; University of Maryland, School of Medicine

**Keywords:** adoption, children, genetics, home environment, human gut microbiome

## Abstract

The composition of the human gut microbiome is highly variable, and this variation has been repeatedly tied to variation in human health. However, the sources of microbial variation remain unclear, especially early in life. It is particularly important to understand sources of early life variation in the microbiome because the state of the microbiome in childhood can influence lifelong health. Here, we compared the gut microbiomes of children adopted in infancy to those of genetically unrelated children in the same household and genetically related children raised in other households. We observed that a shared home environment was the strongest predictor of overall microbiome similarity. Among those microbial taxa whose variation was significantly explained by our models, the abundance of a given taxon was more frequently explained by host genetic similarity (relatedness), while the presence of a given taxon was more dependent upon a shared home environment. This suggests that although the home environment may act as a species source pool for the gut microbiome in childhood, host genetic factors likely drive variation in microbial abundance once a species colonizes the gut.

## Abstract (precis)

Our results demonstrate that the early life home environment can significantly alter the gut microbiome in childhood, potentially altering health outcomes or risk for adverse health outcomes. A better understanding of the drivers of gut microbiome variation during childhood could lead to more effective intervention strategies for overall health starting in early life.

## INTRODUCTION

The high levels of interindividual variation commonly observed in the human gut microbiome (1, 2) potentially arise from host genetic and environmental (i.e., non-host-genetic) factors, as well as their interaction. Potential environmental factors include local conditions, such as variation in hygiene and diet across households. Many epidemiological studies have attempted to identify the relative contributions of host genetic and environmental factors to variation in the human gut microbiome, including factors such as age or life stage (3–5), geographic region (4, 6), or diet (7, 8). However, these factors are often at least partially confounded. For example, studies comparing gut microbiome compositions across geographic regions are important for understanding broad patterns in host-associated microbial diversity; however, human populations are often genetically and culturally stratified across sample locations, leading to unresolvable confounds in genetic and lifestyle-associated factors. Additionally, foundational work in twin cohorts has estimated the overall heritability of the microbiome and identified several heritable taxa by utilizing genetically informed study designs (9–11), but twins reared together during childhood are both related and share a home environment, inextricably linking genetic relatedness and environmental similarity.

We used a sibling adoption study design as a tool to determine the relative impact of host genetic relatedness and shared home environment on the composition of the gut microbiome of children. This design utilizes factorial combinations of genetic relatedness and shared home environment to reduce covariance between these factors, allowing a more rigorous determination of their relative contributions. We compared the gut microbiomes of children adopted in infancy to those of genetically unrelated children in the same household and genetically related children reared in other households. This design also allowed us to identify ecological patterns in the prevalence and abundance of individual microbial taxa in the human gut microbiome.

## RESULTS

### Partitioning variance of whole-community dissimilarity.

**FIG 1:**
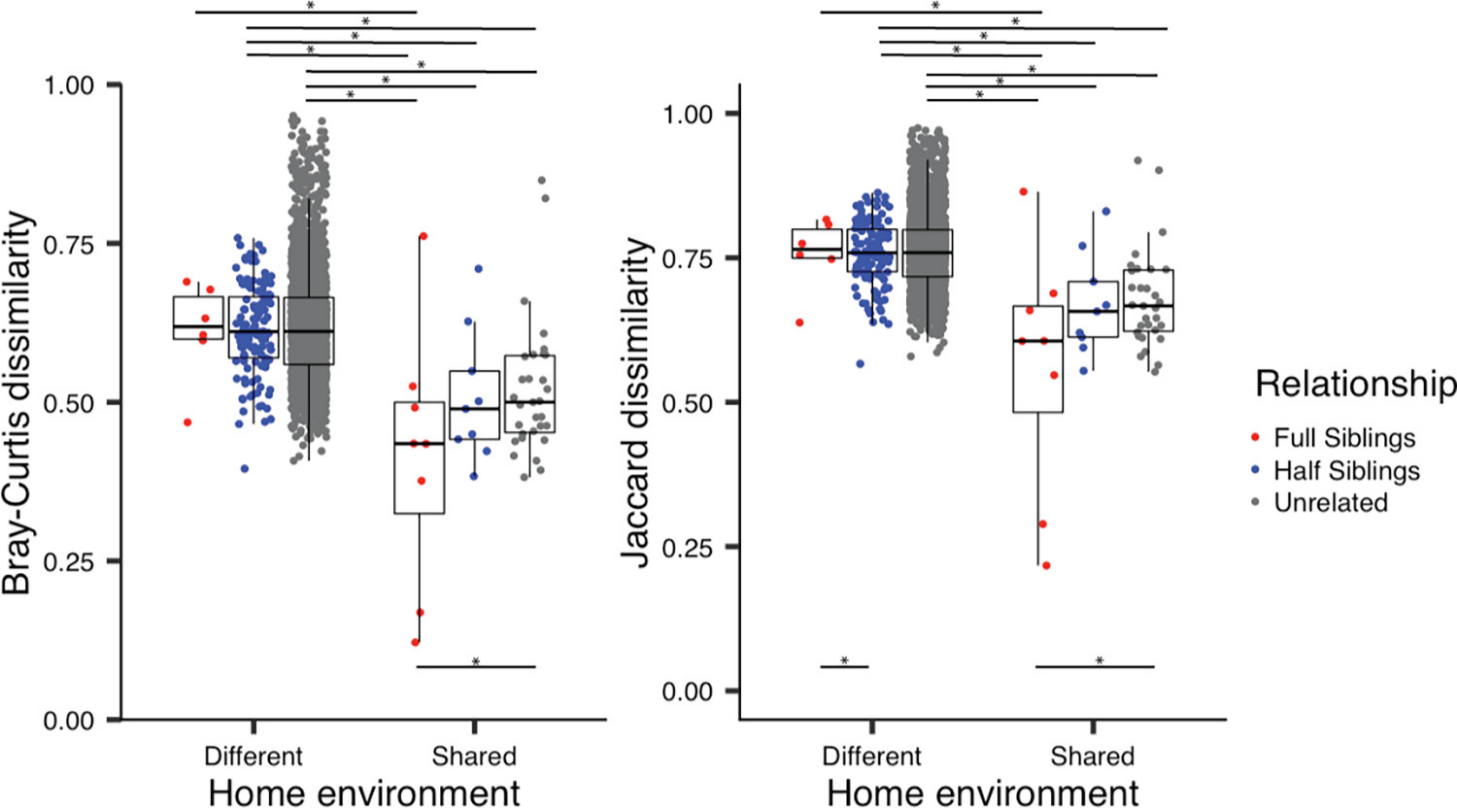
Gut microbiome community dissimilarity by home environment and genetic relatedness. Shown are results for Bray-Curtis dissimilarity (left) and Jaccard dissimilarity (right). The relationship of each child pair is color coded, and observations are clustered by (relationship within) rearing environment. GDM models detected a significant overall effect of shared environment, and any significant pairwise differences in mean dissimilarity metric are denoted among (relationship within) rearing environment groups by an asterisk (adjusted for multiple comparisons and estimated using a Tukey’s honestly significant difference [HSD] test). For each box plot, all data points are plotted: center line indicates the median, box limits indicate the upper and lower quartiles, whiskers indicate 1.5× interquartile range, and points beyond whiskers indicate outliers.

We assessed the relative contributions of host genetic similarity, shared home environment, and multiple host characteristics (sex, age, and body mass index [BMI]) to gut microbiome composition in a cohort of 74 children (mean age, 11.1 years old) from households across the United States. Pairwise values of host genetic similarity were estimated using genomic markers, and pairwise home environment sharing was coded using a binary coding scheme. A single stool sample was collected from each participant, and the microbial community was characterized using 16S-based taxonomic identification. We used the tool “Generalized Dissimilarity Modeling” (GDM [12]) to partition variance in overall microbiome community dissimilarity based on differences in the relative abundance of amplicon sequence variants (ASVs; Bray-Curtis dissimilarity) and differences in the presence/absence of ASVs (Jaccard dissimilarity). Jaccard dissimilarity can be thought of as the proportion of unshared taxa between two individuals. GDM is an extended form of matrix regression, used here to determine the proportion of variance in overall microbiome community dissimilarity that can be explained by pairwise differences in host characteristics, genetics, and home environment (shared or not). GDM models explained 5.15 and 6.19% of total variance in the gut microbial communities among samples using Bray-Curtis and Jaccard dissimilarity metrics, respectively. Home environment, sibling age difference, and human genomic dissimilarity were retained in both abundance and presence models following permutation-based model selection; however, a shared rearing environment was the only predictor contributing significantly to explainable variance in community-level microbiome composition (Table 1 and Fig. 1). Patterns of explained variance were similar when extremely similar pairs were excluded from the analyses (see Table S1 in the supplemental material). Therefore, we can conclude that shared home environment has a detectable influence on gut microbiome composition in childhood, and although host genetic similarity and age improved the overall fit of the GDM model, these factors did not significantly predict microbiome community dissimilarity in this sample of children.

**TABLE 1:** Generalized dissimilarity model output

Dissimilarity metric	Total % of variance explained	Predictor	% of total variance explained	*P* value
Bray-Curtis	5.15	Child age (yr)	35.04	0.172
		Genetic relatedness (*r*)	4.97	0.218
		Home environment (same/different)	40.67	0.050

Jaccard	6.19	Child age (yr)	28.26	0.166
		Genetic relatedness (*r*)	7.03	0.138
		Home environment (same/different)	41.87	0.032

10.1128/mBio.00548-21.2 TABLE S1 Generalized dissimilarity model (GDM) output excluding extreme values. Download Table S1, XLSX file, 0.01 MB. Copyright © 2021 Tavalire et al. 2021 Tavalire et al. https://creativecommons.org/licenses/by/4.0/ This content is distributed under the terms of the Creative Commons Attribution 4.0 International license.

### Partitioning variance of ASV abundance or presence.

**FIG 2:**
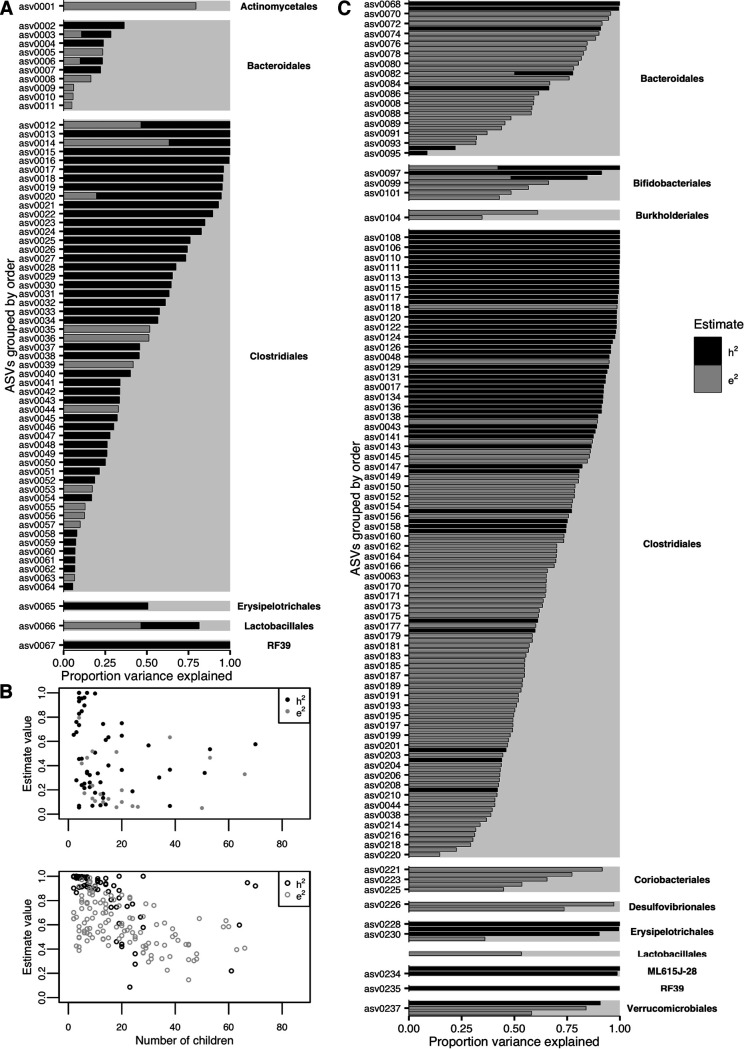
Relative proportion of variance explained by genetics and environment for each significant ASV. (A and C) ASVs whose abundance (A) or presence (C) can be at least partially explained by host genetics (black; *h*^2^) or shared environment (gray; *e*^2^). (B) The magnitude of each variance component estimate relative to how many children’s samples contained that ASV. In the top panel, solid circles represent abundance models, and in the bottom panel, open circles represent presence/absence models. Although some taxa were rare, many taxa whose abundance and presence were significantly predicted by a shared genetic background or home were found at medium to high prevalence in the data set. Alternating ASVs are labeled in panel C. See the supplemental material for full model results.

We used a negative binomial or binomial mixed-effects modeling approach (13, 14) to determine the relative contribution of host genetics and shared home environment to the relative abundance or presence of each ASV, respectively. To estimate the relative contribution of these factors, we included home ID (shared home environment) as a categorical random effect in each model, as well as marker-based estimates of pairwise genome sharing to quantify pairwise host genetic similarity. We identified 67 ASVs whose abundance was significantly explained and 184 ASVs whose presence was significantly explained by either host genetic similarity or environmental similarity, out of 1,813 testable ASVs (i.e., non-singletons detected in at least two samples; grouped by order in Fig. 2 and in Table S2 in the supplemental material). Total read counts across individuals ranged from 7 to 61,350 reads in those ASVs with significant variance components, and these ASVs were found in 2 to 70 children in the data set (Fig. 2B). Ten ASVs from the *Clostridiales* order and 3 ASVs from the *Bacteroidales* order yielded significant variance components in both the abundance and presence/absence models.

10.1128/mBio.00548-21.3 TABLE S2 Variance component model output for explainable ASVs. Download Table S2, XLSX file, 0.1 MB. Copyright © 2021 Tavalire et al. 2021 Tavalire et al. https://creativecommons.org/licenses/by/4.0/ This content is distributed under the terms of the Creative Commons Attribution 4.0 International license.

### Broad-scale patterns.

Among those ASVs whose variation was significantly explained by our models, we characterized patterns in explainable variance by testing if abundance or presence was more frequently explained by host genetic similarity or shared home environment. We found that genetic similarity explained variance in abundance more often than a shared home environment, while presence was more likely explained by a shared home environment (χ^2^ = 24.303, df = 1, *P* = 8.231e−07). These findings are consistent with general ecological principles, which posit that the presence of a species in an ecological community is ultimately due to its availability for dispersal from a regional “species pool” (here, the local home environment), while its abundance may be determined through local environmental selection (here, via individual host genomic factors).

To determine if the presence or abundance of any bacterial orders was more likely to be explained by shared genetics and shared home environment, we tallied original counts of ASVs categorized by order and then used a random permutation test to determine if any orders were oversampled in our results. (The order was the finest level or taxonomic organization for which there were no missing data.) Of the ASVs whose variance could be at least partially explained by host genetics or shared home environment using the mixed-effects modeling approach, those belonging to the *Bifidobacteriales* order were significantly oversampled in the presence models relative to a random sampling from their initial prevalence in the data set (based on 9,999 permutations of 184 ASVs randomly selected out of the total of 1,813; *P* < 0.05). Bacteria of the *Bifidobacteriales* order are common members of a healthy gut microbiome, especially early in life (4), and play a key role in host metabolism and modulating the growth of other bacteria, including pathogens (15). As the presence of members of the *Bifidobacteriales* order is partially predicted by shared genetics and home environment, and given their important role in host health and microbial community dynamics, members of this order could represent a promising intervention target for early life microbiome health.

## DISCUSSION

Overall, we were able to explain a relatively small but significant proportion of the variance in gut microbiome composition in this cohort, using only host genetic similarity and shared home environment as factors. Cross-sectional studies of adult gut microbiomes rarely explain more than 15 to 20% of the variance in composition, even when considering hundreds of factors (although higher levels of variation are often explained in controlled experimental manipulation of diet or antibiotic use [e.g., see references 16 and 17]), and adult gut microbiomes are considerably less variable than those of children (3, 18). For example, a previous study with thousands of participants and hundreds of environmental variables could explain less than 17% of variation in gut microbiome composition (19), while with a single metric of shared environment, we explained more than 6%. This suggests that in childhood, environmental context is largely determined by local rearing environment, within which children share exposures to the natural and built environments, diet, exercise, and hygiene practices. Additionally, we observed that variance in the abundance of individual taxa is more often explained by shared genetics, while the presence of taxa may depend more on environmental context. Aside from these prominent patterns, we also observed that the abundance of some taxa appears to be wholly dependent upon shared home environment, suggesting that variation in exposure mediated by the rearing environment may drive patterns in abundance along with host genetic factors. We also observed that the presence of multiple taxa can be explained wholly by shared genetics and thus that host genetics may still influence the presence of specific bacteria in the gut, regardless of dispersal from the immediate environment.

Our study was not designed to identify the exact components of the rearing environment that contribute to microbiome composition. Future research utilizing repeated, simultaneous sampling of children and their immediate environments, as well as detailed dietary and medical surveys, are necessary to further address this question. Our study also did not consider potential interactions of genetics and environment, or environments experienced by children other than the home. It would be interesting to expand on our study to include other common childhood environments (such as day care centers [20] and schools) or social networks (utilizing family or peer interaction logging [21]). It is possible that inclusion of such information could increase the proportion of microbiome variance explained, approaching the higher levels of variance attributed to shared rearing environment in controlled experiments involving animals (22–24).

Our study demonstrates that adoption in early infancy can result in a measurable shift in the composition of the gut microbiome of children, potentially mediated through the home environment shared with nonrelated siblings. Our results also suggest that the early life home environment may significantly alter the gut microbiome in childhood through differential microbial exposure, posing potentially important consequences for health, both during childhood and later in life (25, 26). Understanding the drivers of gut microbiome variation during childhood could lead to more effective health intervention strategies early in life.

## MATERIALS AND METHODS

### Study subjects.

Participants were part of the Early Growth and Development Study (EGDS [27]) and its companion study, Early Parenting of Children (EPoCh [28]). Together, these studies include a prospective adoption cohort of children who were domestically adopted shortly after birth (median age = 2 days; standard deviation [SD] = 12.45 days, range = 0 to 91 days) into an adoptive home (with unrelated adoptive parents and siblings), the adoptees’ related siblings who remained living with the birth parent(s), and additional unrelated siblings living in either study home.

The subsample for this study was recruited by phone to provide a stool sample, a saliva sample (DNA), and a questionnaire by mail. Participants included 74 children across 26 adoptive and 13 birth homes (39 total households) with different levels of genetic relatedness residing in the same or different homes (8 full sibling pairs reared together, 6 full sibling pairs reared apart, 9 half-sibling pairs reared together, 122 half-sibling pairs reared apart, and 31 nonbiological sibling pairs reared together). All half-siblings were maternal half-siblings (sharing a mother, but with different fathers). The sample was 49% male, and children ranged in age from 4.3 to 18.8 years old at the time of stool collection (mean age = 11.1 years, SD = 3.1 years). We observed no differences in sex ratio (χ^2^ = 1.826, df = 1, *P* = 0.177), age (*t* = −0.097, df = 35.736, *P* = 0.924), or body mass index (BMI [*t* = −1.897, df = 40.034, *P* = 0.065]) between home types in this subsample. Age difference did not vary among sibling types (*F* = 0.1689, *P* = 0.845). Adoptees in this subset were adopted shortly after birth (*n* = 51, mean = 6.4 days, SD = 10.2 days, range = 0 to 48 days); however, one child was adopted at 356 days of age. (This child was not a part of the aforementioned original study sample and was an additional adoptee in an adoptive home with no related siblings in other homes.) This research received approval by the institutional review boards at the University of Oregon, all adult participants provided informed consent, and children provided assent prior to participation.

### Microbiome collection and sequencing.

Stool samples were collected in the home using the OMNIgene-gut fecal collection kit (which contains a nucleic acid preservative) following the kit’s instructions (DNA Genotek; OMR-200) and were returned via standard mail at ambient temperature. Samples were stored at −20°C upon receipt and transferred to −80°C for long-term storage within 4 weeks of receipt, until DNA extraction using the MoBio PowerFecal DNA isolation kit. Each sample was amplified in triplicate using a standard PCR protocol targeting the 16S rRNA variable region V4 (Illumina 515F and 806R). Samples and no-template negative controls were sequenced on the Illumina HiSeq4000 sequencing platform (paired-end 150-bp reads) with a target sequencing depth of 50,000 reads per sample. All read clustering and quality filtering was performed in *QIIME2* (29) using default settings, and the *q2-dada2* pipeline plug-in (30) was used to call individual amplicon sequence variants (ASVs) at 100% sequence similarity based on the 16S rRNA variable V4 region (31). Sequencing depth ranged from 39,523 to 73,295 reads per sample. Analyses of quality and α diversity revealed no effect of transportation or freezing time on overall microbiome Shannon diversity (*r* = −0.04, *P* = 0.720) or read quality (*r* = −0.066, *P* = 0.560).

We identified a total of 3,629 ASVs in stool samples from this study population. A total of 1,588 ASVs were unique to a single sample, and 1,692 ASVs were unique to a single home. A total of 104 of these were shared across stool samples from all children reared in a single shared home environment. Conversely, 1,785 ASVs were found in stool samples from multiple children across multiple home environments. In general, ASVs that were more common across home environments also occurred in more children in the sample, with variation in prevalence across taxonomic groups (see Fig. S1 in the supplemental material). ASVs with fewer than five reads across pooled samples were removed to account for potential sequencing errors or misalignment, leaving 3,055 ASVs for subsequent analyses. α diversity did not differ by home type (Shannon diversity in birth versus adoptive homes: *t* = 0.46901, df = 46.985, *P* = 0.641).

10.1128/mBio.00548-21.1 FIG S1 Distribution of ASVs across children and homes. (A) Reads per child for each ASV over the total number of children whose samples contained that ASV, (B) distribution of ASV prevalence in both children and homes, and (C) prevalence of different ASVs by bacterial order. For each box plot, all data points are plotted: the center line indicates the median, the box limits indicate the upper and lower quartiles, the whiskers indicate 1.5× the interquartile range, and the points beyond whiskers indicate outliers. Download FIG S1, DOCX file, 2.0 MB. Copyright © 2021 Tavalire et al. 2021 Tavalire et al. https://creativecommons.org/licenses/by/4.0/ This content is distributed under the terms of the Creative Commons Attribution 4.0 International license.

### Genomic sequencing and relatedness estimation.

To estimate pairwise relatedness among individuals in the sample, DNA was collected using the Oragene-Discover saliva kit (DNA Genotek; OGR-500). DNA was extracted using the DNAdvance genomic DNA isolation kit (Agencourt; A48705), and individuals were genotyped on the Infinium Global Screening Array-24 v2.0 microarray (Illumina). Genotypes were called and quality filtered using default settings in GenomeStudio (32). We estimated pairwise relatedness using three sets of 2,500 randomly selected single nucleotide polymorphism (SNP) markers from the GSA microarray in the R package *related* (33). The estimated relatedness values were highly correlated across all three subsets (*r* > 0.90 and *P* < 0.001 for all pairwise correlations), and we therefore proceeded with the relatedness estimates calculated using one of the three subsets of markers for subsequent analyses.

### Statistics.

**(i) Partitioning variance of whole-community dissimilarity.** Here, we utilized the ecological statistical tool “Generalized Dissimilarity Modeling (GDM)” in the R package *gdm* (12) as a novel approach to partitioning variance in the gut microbiome community. This model is an extended form of matrix regression in which pairwise community dissimilarity is regressed onto multiple matrices quantifying pairwise dissimilarities in predictor measurements across samples. Total deviance explained is calculated relative to an intercept-only model, analogous to the total variance explained by the full model. Here, we use Bray-Curtis dissimilarity and Jaccard dissimilarity as β diversity metrics based on ASV abundance and presence, respectively. The initial full models for each β diversity metric included pairwise difference in natural log-transformed age, sex (coded as 1 for male and 2 for female), age-corrected BMI Z-score (calculated from height and weight provided at sampling [34]), and matrices of genomic dissimilarity (1 − *r*) and rearing environment dissimilarity (where same home = 0 and different home = 1). Best-fit models were selected based on overall model *P* value, and the relative importance of each predictor was assessed using the recommended permutation methods in the *gdm* package (12) (999 permutations).

**(ii) Partitioning variance of ASV abundance or presence.** In order to determine to what extent variance in abundance and presence of ASVs is explained by genome sharing or a shared home environment, we expanded existing variance partitioning methods (35, 36) in the R package *NBZIMM* (13, 14). ASVs observed in only one child were removed from this analysis since singular observations have no variance, leaving 1,813 testable ASVs. Each negative binomial (abundance) or binomial (presence) model included natural log-transformed age in years and sex as fixed effects and natural log-transformed sequencing depth as an offset term to control for differences in sequencing effort across samples. We then included home and child ID as random effects in each model, using the pairwise relatedness matrix as the correlation structure of the “ID” random effect (35), allowing us to partition the residual variance into that which can be explained by home sharing and genome sharing. We used likelihood ratio testing in the *lmtest* package (37) to assess the significance of each variance component in the model as previously described (35). Any model for which the addition of any variance component resulted in a positive change in log likelihood and that had a false-discovery rate (FDR [38]) corrected *q* value greater than 0.05 was retained for further significance testing. From these models, we eliminated any models in which all variance components were <0.05 in magnitude to focus our analyses on taxa for which appreciable proportions of variance could be explained. We then used a permutation test to create the null distribution for each variance component of each model independently and used these distributions to estimate *P* values for each variance component (based on 499 permutations). Results are aggregated at the order level for ease of interpretation, but all variance partitioning models were run on ASV-level data.

**(iii) Assessing broad-scale patterns.** We hypothesized that factors affecting ASV abundance and presence likely arise from different ecological processes. We therefore determined if ASV abundance and presence were differentially explained by host genetics or shared environment using a χ^2^ test. We totaled the number of significant variance components of each type across significant abundance or presence models and used these values to test for a difference in representation of significant variance components across model types.

We tested the hypothesis that ASVs of certain taxonomic groups may be overrepresented in the list of those whose abundance or presence can be significantly explained by host genetics or host environment by identifying each ASV (*n* = 1,813) to order and randomly selecting the number of significant models from this pool over 9,999 permutations. We then compared counts across orders to the observed counts to determine what proportion of 9,999 random permutations selected the same or more ASVs from a given order to compute a *P* value for each order, describing the extremity of the observed counts relative to random chance. Order was the finest scale of taxonomic organization for which there were no missing data for any ASVs found in more than one sample.

### Data availability.

All relevant processed data supporting the findings of this study are available in the main text or the supplemental material. Unprocessed 16S data are available from the corresponding author upon reasonable request. All code developed for this work builds upon and implements existing R resources and will be provided upon request.

10.1128/mBio.00548-21.4 DATA SET S1 Bray-Curtis dissimilarity matrix. Shown is a symmetric matrix of pairwise values of Bray-Curtis dissimilarity formatted for use in the *gdm* package. Download Data Set S1, TXT file, 0.06 MB. Copyright © 2021 Tavalire et al. 2021 Tavalire et al. https://creativecommons.org/licenses/by/4.0/ This content is distributed under the terms of the Creative Commons Attribution 4.0 International license.

10.1128/mBio.00548-21.5 DATA SET S2 Jaccard dissimilarity matrix. Shown is a symmetric matrix of pairwise values of Jaccard dissimilarity formatted for use in the *gdm* package. Download Data Set S2, TXT file, 0.06 MB. Copyright © 2021 Tavalire et al. 2021 Tavalire et al. https://creativecommons.org/licenses/by/4.0/ This content is distributed under the terms of the Creative Commons Attribution 4.0 International license.

10.1128/mBio.00548-21.6 DATA SET S3 Genetic dissimilarity matrix. Shown is a symmetric matrix of pairwise values of genetic dissimilarity formatted for use in the *gdm* package. This matrix can be altered to report genetic similarity for use in the variance component models by subtracting each value from 1. Download Data Set S3, TXT file, 0.02 MB. Copyright © 2021 Tavalire et al. 2021 Tavalire et al. https://creativecommons.org/licenses/by/4.0/ This content is distributed under the terms of the Creative Commons Attribution 4.0 International license.

10.1128/mBio.00548-21.7 DATA SET S4 Environmental dissimilarity matrix. Shown is a symmetric matrix of pairwise values of environmental dissimilarity formatted for use in the *gdm* package. This matrix can be altered to report environmental similarity for use in the variance component models by subtracting each value from 1. Download Data Set S4, TXT file, 0.01 MB. Copyright © 2021 Tavalire et al. 2021 Tavalire et al. https://creativecommons.org/licenses/by/4.0/ This content is distributed under the terms of the Creative Commons Attribution 4.0 International license.

10.1128/mBio.00548-21.8 DATA SET S5 Predictor variables for GDM. Shown are deidentified individual covariates of sex, BMI (age-corrected Z-score), and age (natural log transformed) used in the GDM models. Download Data Set S5, XLSX file, 0.01 MB. Copyright © 2021 Tavalire et al. 2021 Tavalire et al. https://creativecommons.org/licenses/by/4.0/ This content is distributed under the terms of the Creative Commons Attribution 4.0 International license.

10.1128/mBio.00548-21.9 DATA SET S6 ASV prevalence and taxonomic information. Shown is summary information for each ASV, including the total read count in the entire data set, the total number of children and homes each ASV was detected in, and taxonomic information for each ASV. Download Data Set S6, XLSX file, 0.2 MB. Copyright © 2021 Tavalire et al. 2021 Tavalire et al. https://creativecommons.org/licenses/by/4.0/ This content is distributed under the terms of the Creative Commons Attribution 4.0 International license.

10.1128/mBio.00548-21.10 DATA SET S7 ASV table with covariates for the variance component model. Shown are the deidentified ASV count data for variance component models, including covariates (sex, age, BMI, and sequencing depth) and home IDs. Download Data Set S7, XLSX file, 0.2 MB. Copyright © 2021 Tavalire et al. 2021 Tavalire et al. https://creativecommons.org/licenses/by/4.0/ This content is distributed under the terms of the Creative Commons Attribution 4.0 International license.
