# Supplementary figures and images for: Shared Environment and Genetics Shape the Gut Microbiome after Infant Adoption
Source: mBio. 2021 Mar 30;12(2):e00548-21. doi: 10.1128/mBio.00548-21 (PMC8092250; doi:10.1128/mBio.00548-21)

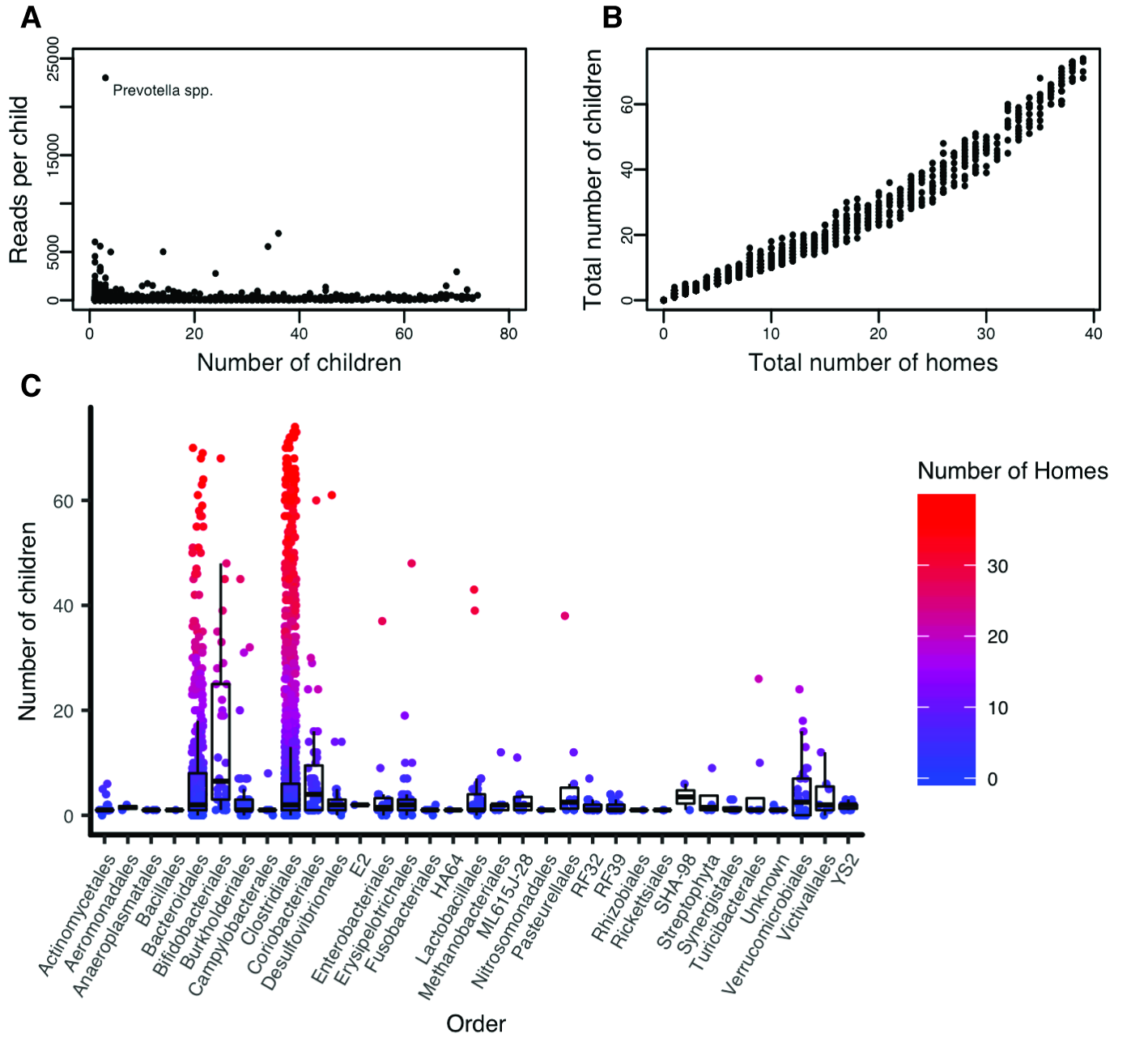

Supplement: FIG S1 [file mBio.00548-21-sf001.docx]
